# Interacting Coastal Based Ecosystem Services: Recreation and Water Quality in Puget Sound, WA

**DOI:** 10.1371/journal.pone.0056670

**Published:** 2013-02-22

**Authors:** Jason Kreitler, Michael Papenfus, Kristin Byrd, William Labiosa

**Affiliations:** 1 Western Geographic Science Center, U.S. Geological Survey, Boise, Idaho, United States of America; 2 Woods Institute for the Environment, Stanford University, Stanford, California, United States of America; 3 Western Geographic Science Center, U.S. Geological Survey, Menlo Park, California, United States of America; 4 Western Geographic Science Center, U.S. Geological Survey, Seattle, Washington, United States of America; MESC; University of South Alabama, United States of America

## Abstract

Coastal recreation and water quality are major contributors to human well-being in coastal regions. They can also interact, creating opportunities for ecosystem based management, ecological restoration, and water quality improvement that can positively affect people and the environment. Yet the effect of environmental quality on human behavior is often poorly quantified, but commonly assumed in coastal ecosystem service studies. To clarify this effect we investigate a water quality dataset for evidence that environmental condition partially explains variation in recreational visitation, our indicator of human behavior. In Puget Sound, WA, we investigate variation in visitation in both visitation rate and fixed effects (FE) models. The visitation rate model relates the differences in annual recreational visitation among parks to environmental conditions, park characteristics, travel cost, and recreational demand. In our FE model we control for all time-invariant unobserved variables and compare monthly variation at the park level to determine how water quality affects visitation during the summer season. The results of our first model illustrate how visitation relates to various amenities and costs. In the FE analysis, monthly visitation was negatively related to water quality while controlling for monthly visitation trends. This indicates people are responding to changes in water quality, and an improvement would yield an increase in the value of recreation. Together, these results could help in prioritizing water quality improvements, could assist the creation of new parks or the modification of existing recreational infrastructure, and provide quantitative estimates for the expected benefits from potential changes in recreational visitation and water quality improvements. Our results also provide an example of how recreational visitation can be quantified and used in ecosystem service assessments.

## Introduction

Coastal and marine ecosystem services – the benefits people derive from marine and coastal ecosystems [1], [2] – are increasingly used in applied marine ecosystem-based management (EBM) and decision making [1], [3], [4], [5]. This group of ecosystem services can include: coastal protection from reefs, vegetation, and dunes [6], [7], [8]; provision of marine products like fish and shellfish [9], [10]; nutrient cycling and waste filtration [11]; recreational opportunities [12]; and cultural values [11], [13]. An understanding of how human actions affect marine ecosystem condition and composition, in the context of EBM and restoration, could guide decisions intended to positively affect the management of ecosystem services and their benefits to human society [5], [14].

In Puget Sound, WA, coastal recreational opportunities and water quality are major contributors to human well-being [13], [15]. Puget Sound tourism and recreation create annual revenues of over $5 billion and provide 62,000 jobs [16], making them important economic contributors. Shellfishing is an important recreational and commercial activity that is regulated based on water quality. The benefits from improved coastal water quality and increased recreation could also compound to produce opportunities for management and restoration actions that would positively affect people, the environment, and the economy. However, the effect of water quality on recreational behavior in Puget Sound has not been quantified, nor have patterns and variation in recreational use been explored for this region. In Southern California, research has addressed the costs of beach closures [17], [18], [19], health effects [20], [21], and public preferences and the value of recreation [17], [18], [22], [23], [24]; these studies provide background for this work, but differences between Puget Sound and Southern California are many, and could lead to different relationships among the factors affecting recreational behavior in Puget Sound.

Studies including recreation as an ecosystem service have used a variety of methods to calculate recreational benefits. Chan et al. [25] used a multicriteria weighted sum to create a GIS value surface of recreational lands, Eigenbrod et al. [26] used survey data to map the popularity of lands for recreational outings, and Hicks et al. [12] and Hein et al. [27] employed the travel cost method (TCM) to value recreation benefits at specific sites. Each technique has benefits and disadvantages. TCM models can value the economic benefits from recreation [28], [29], [30], but rely on visitation data, which is rare in many areas. Map-based approaches that model the complete coverage of recreational benefits across a study area are intuitive and work well in multi-service assessments, but may not be verifiable. By drawing from both approaches, terrestrial and coastal ecosystem service studies could quantify the factors affecting recreational use, and characterize how land use land cover, demographic, or policy changes may influence multiple ecosystem services [5].

To document and clarify the potential effects of water quality and factors affecting recreational use we investigate two datasets: the first contains recreational visits to Washington State Parks that have access to Puget Sound waters, while the second, a water quality monitoring dataset, is analyzed jointly with visitation to determine if environmental condition partially explains variation in recreational visits. Specifically, we address the following questions:

1. What factors explain variation in the recreational use of State Parks within Puget Sound; and
2. What effect does water quality have on recreational visitation?

The answers to these questions provide support for the ecosystem service objectives of the Puget Sound Nearshore Ecosystem Restoration Project (PSNERP - the proposed large scale estuary restoration in Puget Sound [www.pugetsoundnearshore.org]), the Puget Sound Partnership [31](PSP 2008), and for marine and terrestrial recreation components of ecosystem service models and studies [5], [32].

## Methods

### 1.1. Study Area

**Figure 1:**
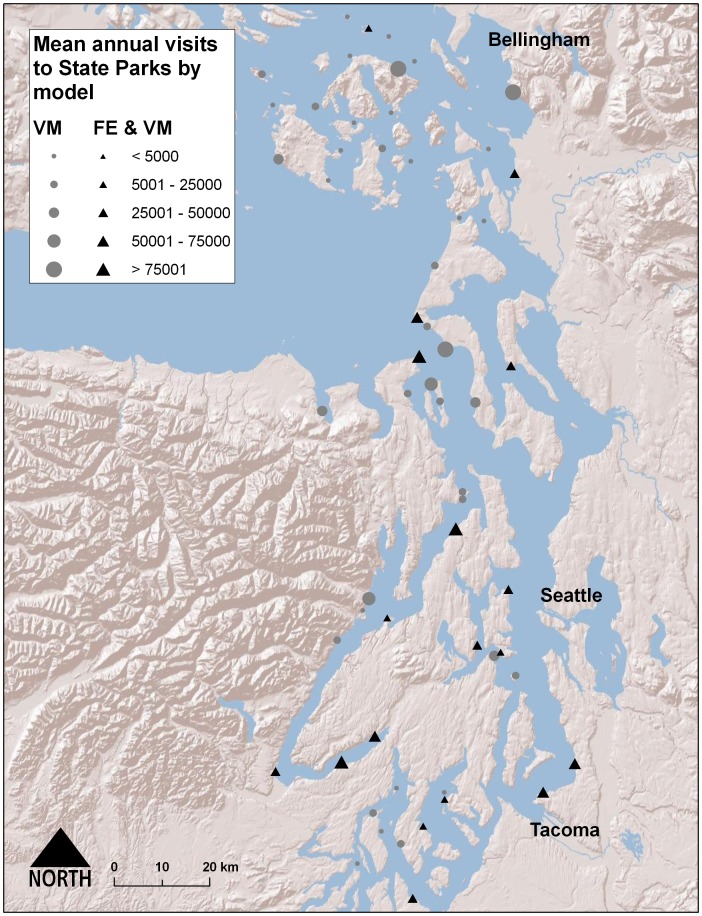
FE and visitation rate model locations and variation in visitors.

The Puget Sound region of Washington, USA, (figure 1) is roughly contained by the watersheds draining into Puget Sound from the Cascade and Olympic Mountains, and the Strait of Juan de Fuca between Washington and Vancouver Island, Canada. This land area of approximately 35,500 km^2^ has a shoreline of nearly 4,000 km, making the coast a prominent feature on the landscape for the region’s 4.4 million inhabitants.

Because much the Puget Sound is deep (450 ft mean depth), the shallow nearshore environment that is most productive and critical for natural ecosystem processes is relatively narrow [16]. The nearshore zone supports many of the marine ecosystem services, including fisheries and recreation, which are characteristic of the Puget Sound region [33]. Historical development patterns have also been centered in this nearshore area due to the maritime roots of Seattle and other nearby communities. Though the region is now a diversified large metropolitan area, the importance of the Sound’s ecosystem services are still apparent to residents: over 500,000 boats are registered in the Sound, 280 marinas are operated in the region, the EPA has designated Puget Sound as an Estuary of National Significance, and studies are underway to begin large-scale ecosystem based restoration projects similar in scope to efforts in the Chesapeake Bay and the Florida Everglades [16].

### 1.2. Data

#### 1.2.1. Visitation

Washington State Parks records visitation numbers based on entrance, camping, and mooring fees. Data are available by month beginning in the late 1980s to mid 1990s to the present, dependent on park. These count data conform to a Poisson distribution through visual inspection, and are simple visitor counts by type to a specific state park and are not distinguished by type of visit (e.g., day use, camping, or mooring). Fifty-seven parks that provide access to the Sound are used in our travel cost method model (table 1); seventeen parks where visitation data and water quality samples were collected concurrently are used for our fixed effects panel model. The requirement of water quality samples and visitation counts leads to an unbalanced panel with 140 observations (Table 2).

**Table 1:** Summary statistics: visitation rate model data.

Variable	Unit	Mean	Std. Dev.
Annual Visits	# Visitor days/year	32,583	42,862
Campsites	#	38.80	47.40
Camping	Dummy	0.75	0.43
Park size	Acres	284	801
Shore length	meters	2,414	3,241
Population availability	# People (eq 1)	29,214	33,422
Travel time	Minutes	129	63
Travel distance	kilometers	76	35
Ferry	Dummy	0.31	0.46
PWC access	Dummy	0.26	0.44
Activities	#	5.90	3.20
Concessions	#	0.73	1.18
Annual ppt	millimeters	926	335
Summer ppt	millimeters	90	12
Sandy	Dummy	0.31	0.46
Heritage	Dummy	0.10	0.30
Shellfishing	Dummy	0.82	0.38
n = 57			

**Table 2:** Summary statistics: fixed effect model data.

	Unit	Obs	Month	Mean	Std. Dev.
Visits	Visitor days	46	June	46,235	43,020
		47	July	65,517	50,308
		47	August	57,237	42,375
Bacteria	#/100 ml	46	June	28.1	41.9
		47	July	23.4	29.7
		47	August	17.6	12.4
Precipitation	mm	46	June	35	11
		47	July	22	21
		47	August	33	36
Observations		140			
Parks		17			
Obs/Park	min	3			
	mean	8.2			
	max	12			

#### 1.2.2. Travel distance and demand

**Figure 2:**
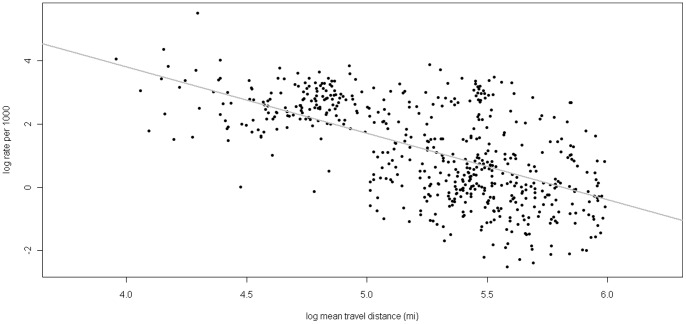
ZIP code visitation rate to Puget Sound State Parks by distance. The natural log of the visitation rate (# visits/1000 population) as a function of the natural log of mean travel distance by zip code (p<0.001, r^2^ = 0.67).

Recreational visitation rates are often a function of site amenities and demand from nearby population centers, with demand typically declining with distance [34], [35]. We model the relationship between distance and visitation rate using an independent dataset from the Washington State parks reservation system containing a visitor’s ZIP code. This dataset is important because it contains the origin location as well as the destination, which the larger visitation dataset lacks. We fit a demand function for visits within 500 miles from Puget Sound parks. Using a road and ferry system dataset and the Network Analyst toolbox in ArcGIS [36], we estimated travel distances for the entire origin/destination matrix. This function relates the visitation rate (# visits/ZIP code population) to the distance traveled (p<0.001, r^2^ = .67, figure 2). We then used this relationship to aggregate demand as a function of distance weighted population around each park using the population availability (PA) method of Coombes et al. [37]:
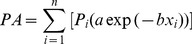
(1)where *n* is the number of US census blocks or Canadian census divisions within the travel distance, *i* is the census unit, *P* is the population size of *i*, *a* is a constant, *b* is the decay coefficient, and *x* is distance. Both *a* and *b* are derived from observed relationship in figure 2. The value of PA for each park is then used as an independent variable in explaining park visitation, and is expected to have a positive sign in the visitation model.

#### 1.2.3. BEACH water quality

**Figure 3:**
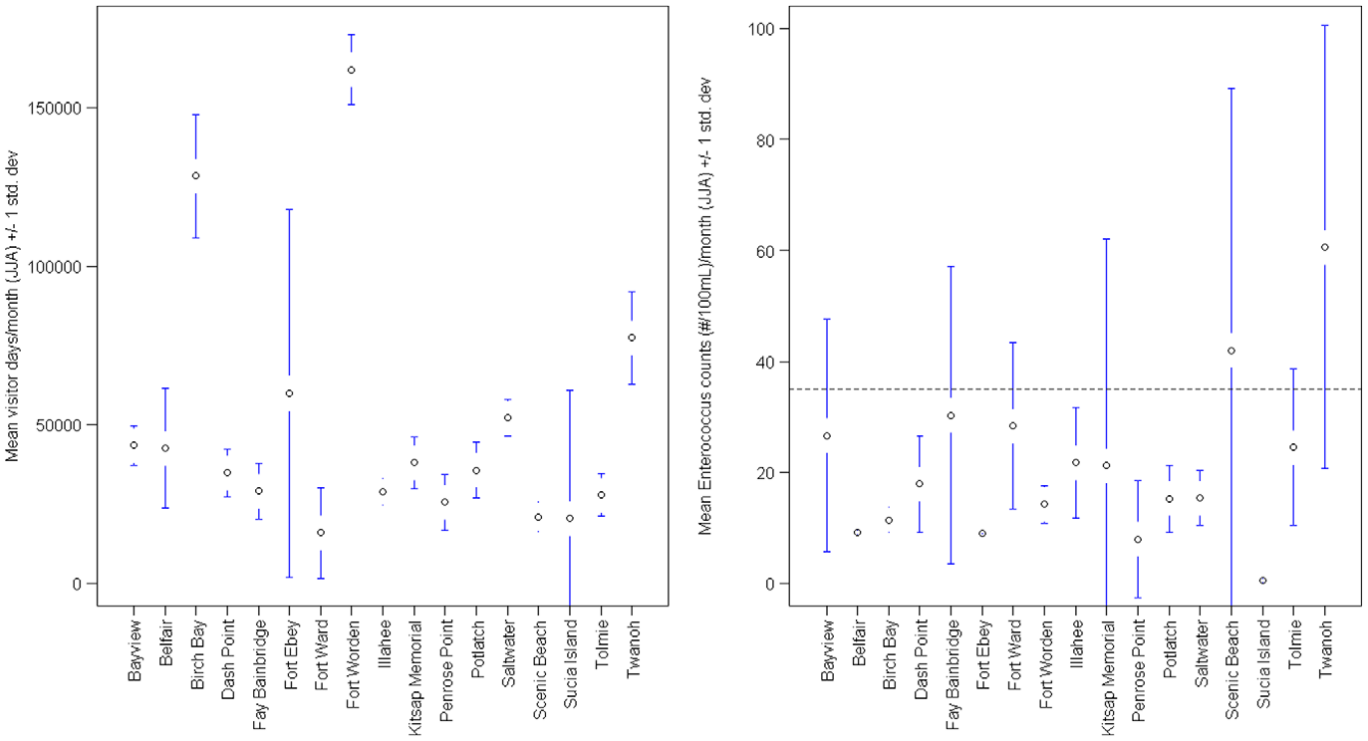
Variation in visitation . Variation in visitation (mean visitor days/month) at the 17 FE model state parks and paired Enterococcus counts from the BEACH program. Dotted line represents the EPA’s marine Enterococcus threshold.

The Washington State Department of Ecology and Department of Health (DOH) monitor water quality in Puget Sound through the EPA’s national Beaches Environmental Assessment and Coastal Health (BEACH) program. *Enterococcus* is tested weekly at coastal recreational swimming beaches during summer months, with water quality results posted on a public website that also notifies users of known pollution events. Beach advisories are issued by counties when bacteria concentrations exceed the EPA threshold, while closures are mainly due to sewage spills or repeated high counts from unknown sources. *Enterococcus* counts exceeded the EPA threshold at seven of the parks we analyzed in this dataset (figure 3).

#### 1.2.4. Other variables

We use several variables to partially explain the variation in visitation. In the fixed effects (FE) model, to control for seasonal, inter-annual, and geographic variations in weather among parks in Puget Sound we use monthly precipitation data by year from the PRISM database [38](PRISM, 2010). In the visitation rate model other explanatory variables included in the model selection process include type of access to the park, park size, beach length, number of listed activities and concessions at each park according to park literature, the number of campsites, and travel time. Travel time to parks from downtown Seattle (which coincidentally is also the population weighted center of the Puget Sound region) was calculated using the same networked travel distance dataset.

### 1.3. Visitation Rate Model

Recreational demand models typically use the travel cost method (TCM) to explain variation in visitation counts [28], [39], [40]. These models assume that the visitation rate to a location depends on the cost of travel from an origin to the destination, socioeconomic factors, and entrance fees. As observed in figure 2, visitation rate increases as travel cost decreases.

We model recreational visitation as a function of park characteristics, travel cost, access, and population availability (Eq 1). Visits are a count variable modeled using the negative binomial distribution [41]. Our data are overdispersed (variance larger than the mean) and without zero counts, thus two models were tested once specified: the negative binomial (NB) and zero-truncated negative binomial (ZTNB) model. This technique addresses the three main problems associated with truncated count data, that they are non-negative integers, cannot have zero values, and are often over-dispersed [42]. We use an information theoretic approach [43] for model selection, and estimate the models in Stata [44].

The models for mean annual park visitation are estimated as:
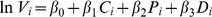
(2)where *V* is the mean annual count of visitors at park *i*, *C* is a vector for the park’s travel cost and access, *P* is a vector of characteristics of each park, and *D* is the population availability surrounding each park.

### 1.4. Fixed Effects (FE) Model

We test the effect of water quality on state park visitation through a fixed-effects panel estimation. The count data are again distributed according to a negative binomial, but in this case we pair repeated monthly visitation counts with *Enterococcus* surveys during summer months to determine the magnitude and direction of the effect of water quality variation. All time invariant heterogeneity among parks is controlled for by this statistical technique [41], leaving changes in visitation to changes in water quality, weather, and time effects. The fixed effects model is estimated as:
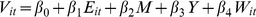
(3)where *V* is the monthly count of visitors at park i in year *t*, *E* is the park’s environmental condition as proxied by the *Enterococcus* counts, *M* is a vector of summer month dummy variables to control for the monthly variation, *Y* is a vector of year dummy variables to control for interannual variation, and *W* is the time-variant mean monthly weather conditions. Equation (2) is estimated using the fixed-effects negative binomial panel model in Stata [44], with reference to a June, 2004 baseline (June and 2004 dropped to avoid multicolinearity).

## Results

### 2.1 Visitation Rate Model

Results from our model can be seen in table 3. Using the 57 State Parks in the dataset, our model explains nearly 70% of the null deviance in mean annual park visitation. Out of the variables initially analyzed, six were retained in the best model through an information theoretic approach [43]. The variables that increased visitation include the number of campsites at a park, the park size, and the number of possible activities at the park. Variables that negatively affected visitation include a dummy variable describing accessibility limited to private non-commercial watercraft, the population availability (equation 1), and the travel time to a park from Seattle. The negative binomial and zero-truncated negative binomial performed almost identically, with only small differences among coefficients and between chi^2^ statistics.

**Table 3:** Visitation rate model results (equation 2).

		NB model				ZTNB model		
Variables	Coefficients	Std. error	*z*	*p*	Coefficients	Std. error	*z*	*p*
campsites	0.0078	0.0028	2.75	0.006	0.0078	0.0028	2.75	0.006
ln(acres)	0.2232	0.0717	3.11	0.002	0.2232	0.0717	3.11	0.002
activities (#)	0.1332	0.0450	2.96	0.003	0.1332	0.0450	2.96	0.003
PWC access (dummy)	−1.6565	0.3036	−5.46	0.000	−1.6565	0.3036	−5.46	0.000
ln(PA)	−0.4482	0.1420	−3.16	0.002	−0.4482	0.1420	−3.16	0.002
ln(travel time)	−0.9147	0.3174	−2.88	0.004	−0.9147	0.3174	−2.88	0.004
cons	16.7207	2.6793	6.24	0.000	16.7208	2.6794	6.24	0.000
Dependent variable = mean annual park visitation								
n =	57				57			
LR chi2	75.28				75.08			
prob>chi2	0.000				0.000			

### 2.2 FE Model

The fixed-effects model results can be seen in table 4. The coefficient of the natural logarithm of the water quality variable, mean *Enterococcus* counts, indicates a 10% increase will decrease visitation to state parks by 2.5%. The model controls for time-invariant factors that could be affecting visitation counts other than water quality. Month and year effects (except 2005) are significant, while weather effects were insignificant; thus we rule out the potential effect of poor weather contributing to decreased visitation. The month dummy variables for July and August are both positive and have roughly the same coefficient value, indicating they exert a similar increase in visitation in reference to the month of June when conditions are cooler and the summer travel season has just begun. Year effects, with reference to 2004, follow prevailing downward economic conditions, and control for general financial factors among years that may influence visitation.

**Table 4:** Response of visits to water quality variation (equation 3).

Variable	Coefficient	Std. error	z	*p*	
ln(count)	−0.2565	0.0631	−4.0600	0.0000	
ppt	−9.62E−06	1.55E−05	−0.6200	0.5340	
June	(dropped)				
July	0.5571	0.1016	5.4800	0.0000	
Aug	0.5414	0.0986	5.4900	0.0000	
yr2004	(dropped)				
yr2005	−0.1644	0.1095	−1.5000	0.1330	
yr2006	−0.2597	0.1207	−2.1500	0.0310	
yr2007	−0.4156	0.1223	−3.4000	0.0010	
cons	1.9905	0.2307	8.6300	0.0000	
Dependent variable = monthly park visitation					
n =	140				
Wald chi^2^	87.39				
prob >chi^2^	0.0000				

## Discussion

When assessing recreation as an ecosystem service [2], [25], visitation is the measure most commonly used to model and quantify the variation of this service. Globally, trends in tourism related to outdoor recreation and wildlife viewing are increasing [45], with recognition that ecosystem services play an important role in generating revenue for conservation and local development [46], [47], [48]. We used the visitation rate model to understand the factors affecting the regional pattern of recreational visitation, and the FE model to determine how water quality can affect visitation at Puget Sound State Parks. These results could be used in planning for restoration and water quality improvement decisions in Puget Sound, and give an example of how GIS analysis and visitation modeling can give a thorough understanding of recreational behavior for use in ecosystem service assessments.

The factors affecting visitation to Puget Sound state parks are similar to other studies that have analyzed recreational behavior in coastal [49], [50] and terrestrial areas [34], [51]. The visitation rate model variables that performed as expected include park size, travel distance, campsites, amenities, and access. All sites within our study had public access, but some require personal watercraft (typically kayaks or small boats) for travel to smaller islands or secluded coastlines without land or ferry access. The size, number of amenities, and number of campsites are important variables in this context because our data contained general visitation counts that were not stratified by specific recreational activities. Therefore we would expect larger parks with a greater variety of potential activities to attract a larger number of visits. Travel time negatively affected visitation, and was measured from downtown Seattle to each park. We used this as a proxy for actual travel time from origin to park destination because origins were not recorded in the larger State Park dataset. Nonetheless, this travel cost measure likely captures the variation in trip length to parks using the road and ferry network in Puget Sound, and is a large improvement over previous methods [52].

Contrary to our *a priori* expectations, PA (eq 1) had a significant negative relationship with visitation. This is likely due to the activity and purpose of a State Park visit, where a preference for a more natural or semi-natural settings away from urbanized areas could be expected based on local values. If our dataset included frequently used urban parks and coastal access points we might have found less of a negative effect. Therefore the type of recreational activity should be carefully considered when analyzing recreational use, particularly if certain activities may not be desirable in all locations. Similarly, we could be observing visitation displacement (or crowd avoidance, [18], [53]) by park visitors. These results indicate a need to collect and analyze visitation data at other points on the Sound to compare recreational use across the entire study area.

**Figure 4:**
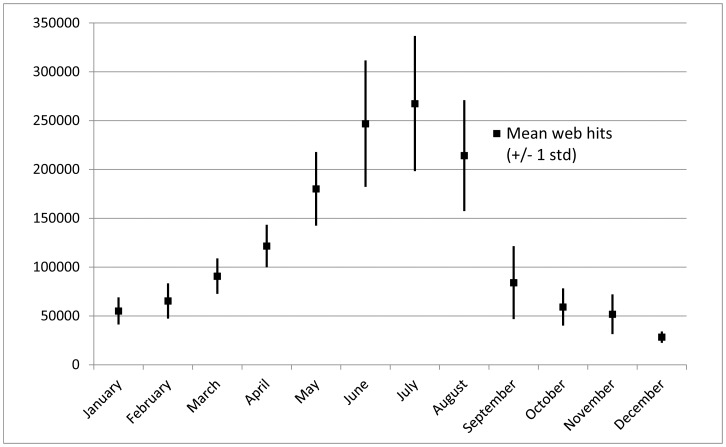
Monthly web hits. Monthly web hits (mean +/−1 st dev) to the WA BEACH program water quality map from 2005–2010.

In the fixed-effects model we show that increasing *Enterococcus* counts negatively affect the number of state park visits made in Puget Sound through a revealed preference approach. Swimming, shellfishing, tide pooling, and other recreational activities with water contact are primary activities at these state parks, so it is understandable that decreased water quality would affect visitation. Of the 50 beaches that DOH monitors as part of the BEACH program within Puget Sound, seven failed to meet EPA water quality standards greater than 8% of the time between 2004–2009 [54]. Our sample of parks contains four that failed the BEACH standard greater than 4% of the time, while the standard deviation of *Enterococcus* counts at seven parks overlapped the EPA marine threshold of 35/100 mL (figure 2). Water quality monitoring data has been available since 2003 through a map-based web user interface from the DOH, and has received an average of 170,000, 186,000, and 147,000 web hits for the months of June, July and August, respectively (figure 4). Though survey evidence of actual beach users would be a more definitive source, the number of web hits to the DOH site supports the explanation that variation in visitation could be from recreational avoidance of higher bacterial counts.

The response of recreational behavior to water quality and beach advisories is mixed in the literature. Recreationalists, in general, respond to water quality but not necessarily beach advisories, though there are few studies documenting either. Busch [21] found surfers in California reduced their exposure to poor water quality by following the “72 hour rule” (avoiding water contact for 72 hrs after a rain event), rather than heeding posted beach advisories. Similarly, a study in San Diego, California found beach advisories did not affect recreational site choice, even though survey respondents ranked water quality the highest factor affecting beach experiences [17]. Hanemann et al., [22] and Hanley et al. [50] found a negative response of beach goers to declining water quality, but only Busch [21] was a true longitudinal revealed preference study. This research adds to the relatively few studies attempting to assess the effect of water quality on recreational visitation, and is the only study available analyzing Puget Sound.

**Figure 5:**
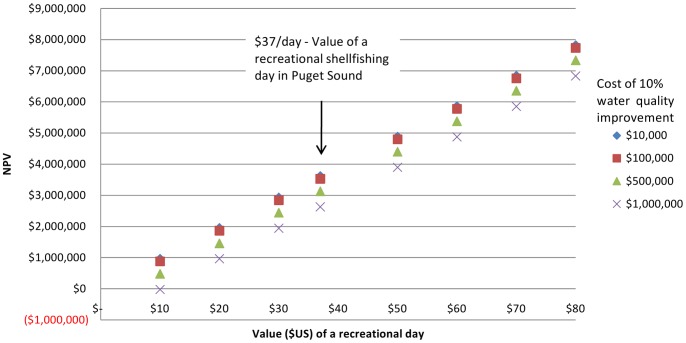
NPV of recreational benefits at Twanoh SP for water quality improvement costs and recreation day values. Net present value calculations assume a 3% discount rate, a 2.5% increase in average summer visitation due to a 10% water quality improvement, and a 25 year time horizon.

The two models presented in this study were developed for potential Puget Sound wide restoration activities by PSNERP and the PSP. In Puget Sound there is limited documentation of recreational behavior available, yet these two organizations have objectives that incorporate increasing recreational opportunity. In deciding how to allocation restoration efforts, Puget Sound restoration and cleanup priority would not necessarily proceed from areas with the poorest to best water quality, but rather to areas where the greatest net benefits (increased ecosystem services) would occur after restoration actions. In some areas large restoration costs may be warranted. For example, using the mean monthly Twanoh State Park visitation, a 3% discount rate [55], and a 25 year time horizon, the net present value of recreational benefits after a 10% water quality improvement could yield positive economic benefits for all but one of the improvement cost/recreational day value combinations (figure 5). Using a local estimate for the value of a recreational shellfishing day in Puget Sound ($37 [56]), the benefit/cost ratio of a 10% water quality improvement costing $1 million would be 2.6∶1, solely through the value of recreation. Documenting the other ecosystem services is therefore critical to determine where actions could affect bundles of ecosystem services such as commercial and recreational fishing and shellfishing, coastal protection and erosion control, and cultural values [5], [57]. The USGS Puget Sound Ecosystem Portfolio Model [58] is a first step in that effort.

Our methods are not without some admitted shortcomings, however. Our visitation data are an aggregated count of visitor days, thus the actual number of trips are likely less than what we report due to multi-day trips. Similarly, people may be taking multi-day trips to multiple parks, which would overestimate our travel cost estimates. These are known critiques of the travel cost method [29], [52], [59]. We use a single origin (Seattle) to calculate travel costs, which is an acknowledged simplification, due to the absence of origins in our main visitation dataset. Had we employed the reservation dataset (as in the PA variable, figure 2) to estimate a unique travel distance for each park our results might be slightly different. In spite of these small factors we believe this study represents a step forward in quantifying the recreational benefits for ecosystem service studies, particularly for the Puget Sound region.

Ultimately it will likely be most cost-effective to consider the value of ecosystem services when prioritizing restoration actions in Puget Sound. Clean water has clear economic benefits that have been previously addressed [60], [61], and in this study we illustrate the positive effect of water quality on the value of recreation. Other values directly tied to water quality, but not present in this study, include commercial and recreational fish and shellfish harvesting, tribal shellfish harvest, residential land values, and other direct and indirect uses [1], [4], [57]. The ecological functions in Puget Sound have many threats facing them in light of climate and land use change, but linking ecology and human behavior for coastal ecosystem based management [62] is one way restoration could be effective in enhancing human well-being in the Puget Sound region and beyond.
